# Characterizing the outcomes of metastatic papillary renal cell carcinoma

**DOI:** 10.1002/cam4.1048

**Published:** 2017-04-16

**Authors:** John Connor Wells, Frede Donskov, Anna P. Fraccon, Felice Pasini, Georg A. Bjarnason, Benoit Beuselinck, Jennifer J. Knox, Sun Young Rha, Neeraj Agarwal, Isaac Alex Bowman, Jae‐Lyun Lee, Sumanta K. Pal, Sandy Srinivas, Douglas Scott Ernst, Ulka N. Vaishampayan, Lori A. Wood, Robin Simpson, Guillermo De Velasco, Toni K. Choueiri, Daniel Y. C. Heng

**Affiliations:** ^1^ Tom Baker Cancer CentreUniversity of CalgaryCalgaryAlbertaCanada; ^2^ Queen's UniversityKingstonOntarioCanada; ^3^ Aarhus University HospitalAarhusDenmark; ^4^ Casa di Cura PederzoliPeschiera Del GardaItaly; ^5^ Medical Oncology DepartmentOspedale Santa Maria della MisericordiaRovigoItaly; ^6^ Sunnybrook Odette Cancer CentreTorontoOntarioCanada; ^7^ University Hospitals LeuvenLeuvenBelgium; ^8^ Princess Margaret HospitalTorontoOntarioCanada; ^9^ Yonsei University College of MedicineSeoulSouth Korea; ^10^ Huntsman Cancer InstituteUniversity of UtahSalt Lake CityUtah; ^11^ University of Texas Southwestern Medical CenterDallasTexas; ^12^ Asan Medical CentreUniversity of Ulsan College of MedicineSeoulSouth Korea; ^13^ City of Hope Comprehensive Cancer CenterDuarteCalifornia; ^14^ Stanford Cancer CenterStanfordCalifornia; ^15^ London Health Sciences CenterLondonOntarioCanada; ^16^ Karmanos Cancer InstituteDetroitMichigan; ^17^ Queen Elizabeth II Health Sciences CentreHalifaxNova ScotiaCanada; ^18^ Dana Farber Cancer InstituteBostonMassachusetts

**Keywords:** Metastatic renal cell carcinoma, outcomes, papillary, response rate, survival, targeted therapy

## Abstract

Outcomes of metastatic papillary renal cell carcinoma (pRCC) patients are poorly characterized in the era of targeted therapy. A total of 5474 patients with metastatic renal cell carcinoma (mRCC) in the International mRCC Database Consortium (IMDC) were retrospectively analyzed. Outcomes were compared between clear cell (ccRCC;* n *= 5008) and papillary patients (*n* = 466), and recorded type I and type II papillary patients (*n* = 30 and *n* = 165, respectively). Overall survival (OS), progression‐free survival (PFS), and overall response rate (ORR) favored ccRCC over pRCC. OS was 8 months longer in ccRCC patients and the hazard ratio of death was 0.71 for ccRCC patients. No differences in PFS or ORR were detected between type I and II PRCC in this limited dataset. The median OS for type I pRCC was 20.0 months while the median OS for type II was 12.6 months (*P *= 0.096). The IMDC prognostic model was able to stratify pRCC patients into favorable risk (OS = 34.1 months), intermediate risk (OS = 17.0 months), and poor‐risk groups (OS = 6.0 months). pRCC patient outcomes were inferior to ccRCC, even after controlling for IMDC prognostic factors. The IMDC prognostic model was able to effectively stratify pRCC patients.

## Introduction

Renal cell carcinoma (RCC) is frequently divided histologically into clear cell (ccRCC) and nonclear cell RCC. ccRCC is the most prevalent form of RCC, accounting for 75–80% of cases, while papillary RCC (pRCC) is the most common variant of nonclear cell RCC, constituting 10–15% of all RCCs 1, 2. pRCC is a heterogeneous disease that can be further divided into two main subtypes based on histopathologic features. Type I tumors typically consists of thin, basophilic papillae cells, whereas type II pRCC have thicker papillae and an eosinophilic cytoplasm 3. Unlike ccRCC, pRCC is not associated with aberrations of the *VHL* gene. Instead, type I pRCC frequently displays alterations of the *MET* gene, whereas type II tumors are associated with alterations of the NRF2‐ARE pathway and the fumarate hydratase gene 4, 5. Some type II pRCC tumors may also exhibit increased *MET* expression 5.

Patient outcomes for pRCCs are not well documented, especially when treated with targeted therapy. Localized pRCC may have a better prognosis than localized ccRCC, but several large studies have reported conflicting results 1, 6, 7, 8. Outcomes for metastatic pRCC patients are even less well characterized, but several smaller studies suggest that ccRCC histology is more favorable than pRCC histology 9, 10. The past decade has seen the treatment of metastatic RCC shift from cytokine‐based immunotherapy to targeted therapies, however, the major clinical trials leading to this paradigm shift primarily included ccRCC patients. Given the distinct genetic differences between pRCC and ccRCC, it is not surprising that therapies targeting the vascular endothelial growth factor (VEGF) and the mammalian target of rapamycin (mTOR) have failed to benefit pRCC patients to the same extent as ccRCC patients 9. To date, there is no standard treatment for pRCC.

The outcomes for metastatic type I and type II pRCC are poorly understood and the majority of clinical trials including pRCC patients often do not distinguish between the subtypes. This study was designed to retrospectively determine the outcomes of metastatic pRCC patients as compared to ccRCC patients treated with targeted therapies, and to compare the outcomes of metastatic type I and type II disease. To the best of the author's knowledge, this is the largest analysis of metastatic pRCC and its subtypes to date.

## Materials and Methods

### Patient population and histology

Twenty‐seven international cancer centers in Canada, the USA, Denmark, Greece, South Korea, Australia, New Zealand, Japan, Singapore, Belgium, and Italy provided consecutive patient data collected from hospital and pharmacy records using uniform database software and templates. Data were collected between 2005 and May 2016. Institutional review board approval was obtained from each participating center.

All patients were diagnosed with mRCC and were treated with at least one approved VEGF (sunitinib, sorafenib, pazopanib, bevacizumab, or axitinib) or mTOR‐targeted therapy (temsirolimus or everolimus). Only patients diagnosed with clear cell or papillary histology were included. Tumor histology was recorded in the data collection template using pathology reports from each respective institution. These pathology reports were completed by pathologists prior to and independently from this study as a part of routine diagnosis. Subtypes were only recorded as type I or type II if explicitly stated on the pathology report. Some tumors were recorded as mixed type I/II histology. Reports that could not differentiate the subtype were recorded as not otherwise specified (NOS). Patients with the subtype unavailable were coded as not available.

### Outcomes

The primary outcome was overall survival (OS) from date of initiation of targeted therapy, while secondary outcomes included progression‐free survival (PFS) and response rate (RR). OS was defined as the time from initiation of targeted therapy to death or censored at last follow up. PFS was defined as the time from initiation of targeted therapy until progression‐based on Response Evaluation Criteria in Solid Tumors (RECIST) guidelines, cessation of therapy, death while on therapy, or censored at last follow‐up 11.

The median OS associated with each first‐line therapy was reported for pRCC patients. Additionally, VEGF and mTOR therapies were pooled separately to compare pRCC response to each drug class based on OS, PFS, and ORR.

To determine the utility of the International mRCC Database Consortium (IMDC) prognostic model in pRCC, patients were stratified into risk groups based on the IMDC prognostic factors: hemoglobin below the lower limit of normal (LLN), corrected calcium greater than the upper limit of normal (ULN), neutrophils above ULN, platelets above ULN, Karnofsky performance status (KPS) below 80%, and time from diagnosis to treatment of <1 year 12. Patients with none, 1 or 2, and 3 or more prognostic factors are categorized as favorable, intermediate, and poor risk, respectively.

### Statistical analysis

Statistical analyses were performed with SAS version 9.4 (Cary, NC). Patient outcomes were compared between ccRCC and pRCC. A further analysis of pRCC was performed to compare outcomes of type I and type II pRCC. Kaplan–Meier curves were constructed to estimate median OS and PFS; these outcomes were compared using the log‐rank test. Cox regression modeling was performed for OS for pRCC versus ccRCC, adjusted using the individual IMDC prognostic factors. Missing data were handled conservatively with the case deletion method. Best achieved RR was reported as complete response (CR), partial response (PR), stable disease (SD), and progressive disease (PD) as based on RECIST guidelines. CR and PR were pooled together as an overall response rate (ORR), which was then used to compare between treatment groups using Fischer's exact test.

## Results

Data from 5474 patients from the IMDC were retrospectively analyzed. Our analysis included 5008 patients with clear cell histology and 466 with papillary histology. Of the 466 pRCC histologies, 30 (6.4%) were type I, 165 (35.4%) were type II, 47 (10.1%) had mixed type I/type II features, 146 (31.3%) were not specified, and 78 (16.7%) had missing information on subtype. (Table 1) Considering only known and recorded subtype histologies (*n *= 242), 11.5% were type I, 68.2% were type II, and 19.4% were mixed histology. ccRCC and pRCC patient characteristics are listed in Table 1.

**Table 1:** Baseline characteristics of ccRCC (clear cell renal cell carcinoma) and papillary renal cell carcinoma (pRCC) patients

Baseline characteristics	ccRCC *n* (%)	pRCC *n* (%)
Male	3685/5008 (73.6%)	366/466 (78.5%)
Nephrectomy	4134/4999 (82.7%)	384/466 (82.4%)
IMDC prognostic group		
Favorable	783/3919 (20.0%)	43/359 (12.0%)
Intermediate	2165/3919 (55.2%)	200/359 (55.7%)
Poor	971/3919 (24.8%)	116/359 (32.3%)
Histology		
Type I	N/A	30/466 (6.4%)
Type II		165/466 (35.4%)
Mixed		47/466 (10.1%)
NOS		146/466 (31.3%)
Data unavailable		78/466 (16.7%)
KPS <80	949/4604 (20.6%)	96/419 (22.9%)
Diagnosis‐to‐treatment <1 year	2617/5003 (52.3%)	280/466 (60.1%)
Hemoglobin <LLN	2502/4677 (53.5%)	272/425 (64%)
Platelet >ULN	716/4554 (15.7%)	86/423 (20.3%)
Neutrophil >ULN	651/4553 (14.3%)	91/418 (21.8%)
Calcium >ULN	597/4306 (13.9%)	47/390 (12.1%)
>1 Metastasis	3711/4930 (75.3%)	344/463 (74.3%)
Brain metastasis	398/4860 (8.2%)	16/441 (3.6%)

NOS, not otherwise specified; KPS, Karnofsky performance status; LLN, lower limit of normal; ULN, upper limit of normal.

OS, PFS, and RR all favored ccRCC over pRCC (Table 2). Median OS was over 8 months longer in ccRCC patients, 21.9 months, as compared to 13.8 months in pRCC. When adjusted by the individual IMDC prognostic factors by Cox regression modeling, the hazard ratio of death for overall survival was 0.71 (95% CI 0.62–0.81, *P* < 0.0001) for patients with ccRCC versus patients with pRCC. PFS was 7.3 months in ccRCC and 4.7 months in pRCC. ORR to first‐line therapy of any type was 30.6% in ccRCC and 10.3% in pRCC (*P* < 0.0001). PD was observed as the best response in 24.9% of ccRCC patients and 34.3% of pRCC patients.

**Table 2:** Outcomes in response to targeted therapy for ccRCC (clear cell renal cell carcinoma) and papillary renal cell carcinoma (pRCC)

	ccRCC (*n* = 5008)	pRCC (*n* = 466)	*P*‐value
OS (months; 95% CI)	21.9 (20.9–22.9)	13.8 (12.5–16.1)	0.0001
PFS (months; 95% CI)	7.3 (6.9–7.7)	4.7 (4.1–5.2)	0.0001
RR to 1st Line TT	*n* (%)	*n* (%)	<0.0001
Complete response	110/4389 (2.5%)	3/391 (0.8%)	
Partial response	1234/4389 (28.1%)	37/391 (9.5%)	
Stable disease	1950/4389 (44.4%)	217/391 (55.5%)	
Progressive disease	1095/4389 (24.9%)	134/391 (34.3%)	
OS by IMDC prognostic group (months; 95% CI)			
Favorable	41.9 (38.0–44.8) (*n* = 783)	34.1 (18.6–49.1) (*n* = 43)	0.40
Intermediate	24.0 (22.8–25.1) (*n* = 2165)	17.0 (13.4–18.7) (*n* = 200)	0.0001
Poor	7.1 (6.5–8.0) (*n* = 971)	6.0 (4.1–7.9) (*n* = 116)	0.03

OS, overall survival; PFS, progression‐free survival; RR, response rate; TT, targeted therapy; IMDC, International mRCC database consortium; CI, Confidence interval.

**Figure 1:**
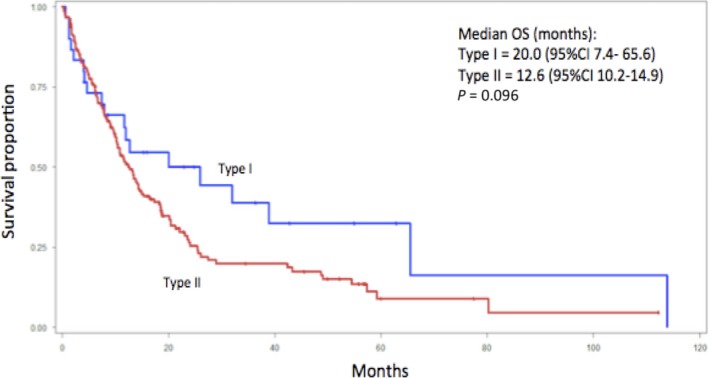
Kaplan–Meier curve depicting the overall survival of type I (*n* = 30) and type II (*n* = 165) metastatic papillary renal cell carcinoma patients treated with targeted therapy.

**Figure 2:**
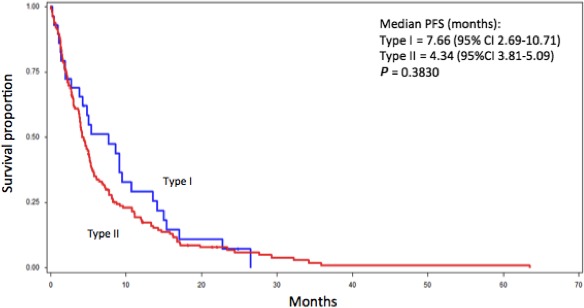
Kaplan–Meier curve depicting the progression‐free survival of type I (*n* = 30) and type II (*n* = 165) metastatic papillary renal cell carcinoma patients treated with targeted therapy.

The median OS for type I pRCC was 20.0 months while the median OS for type II was 12.6 months (*P *= 0.096) (Fig. 1). No differences in PFS were observed for type I and type II (Fig. 2). ORR to any targeted therapy was 9.1% (2/22) for type I patients and 14.3% (20/139) for type II.

For first‐line treated pRCC patients, sunitinib was the most frequently used targeted therapy (63.9%), followed by sorafenib (12.9%), temsirolimus (12.4%) and everolimus (5.1%) (Table 3). No single targeted therapy appeared to provide a superior OS benefit. pRCC outcomes for OS and PFS were similar in patients treated with mTOR inhibitors and VEGF inhibitors (Table 3). However, ORR favored VEGF therapies (11.9%) over mTOR therapies (3.2%, *P *= 0.0413).

**Table 3:** Outcomes in papillary renal cell carcinoma (pRCC) patients treated with first‐line targeted therapy

First‐line therapy (*n*, %)	OS (months, 95% CI)
Sunitinib (288/451 63.9%)	14.9 (12.6–18.2)
Sorafenib (58/451, 12.9%)	11.7 (8.1–20.9)
Bevacizumab (5/451, 1.1%)	16.7 (14.0–20.4)
Temsirolimus (56/451, 12.4%)	11.8 (7.8–14.5)
Everolimus (23/451, 5.1%)	26.0 (8.0–41.7)
Pazopanib (21/451, 4.7%)	11.5 (3.1–14.9)

Outcome	mTOR inhibitors	VEGF inhibitors	*P*‐value
OS (months; 95% CI)	12.4 (8.9–18.2) (*n* = 77)	13.9 (12.5–16.7) (*n* = 377)	NS
PFS (months; 95% CI)	3.7 (2.7‐5.3) (*n* = 76)	4.9 (4.2‐5.4) (*n* = 378)	NS
ORR (CR + PR)	2/63 (3.2%)	38/320 (11.9%)	*P* = 0.0413

OS, overall survival; PFS, progression‐free survival; ORR, overall response rate; CR, complete response; PR, partial response; NS, not statistically significant; CI, confidence interval; mTOR, mammalian target of rapamycin; VEGF, vascular endothelial growth factor.

**Figure 3:**
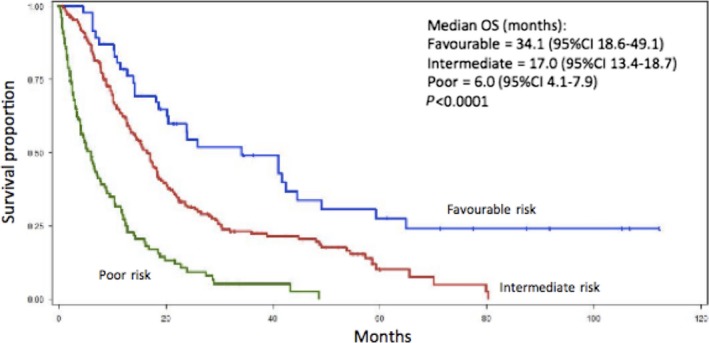
Kaplan–Meier curve depicting the overall survival of 359 metastatic papillary renal cell carcinoma stratified by International mRCC Database Consortium‐risk factor group. Blue = favorable risk (12.0%), Red = intermediate risk (55.7%), Green = poor risk (32.3%).

The IMDC prognostic model stratified pRCC patients into significantly different prognostic groups (*P *< 0.0001). Favorable‐risk patients (0 factors) had an OS of 34.1 months, intermediate‐risk patients (1–2 factors) had an OS of 17.0 months, while poor‐risk patients (three or more factors) had an OS of 6.0 months (Fig. 3).

## Discussion

Until recently, the majority of data regarding pRCC has come from smaller retrospective analyses, subanalysis of clinical trials, expanded access trials, and phase II trials that combined pRCC with other nonclear cell RCCs 9, 13, 14, 15, 16, 17. The IMDC previously reported that metastatic pRCC has a worse outcome than ccRCC, a finding that we have confirmed in a sample size nearly three times larger 9. This gap is likely to increase as further therapies are approved for ccRCC 18. Our study was also able to analyze pRCCs by subtype and report that OS in type I pRCC may be similar to ccRCC, but that a high degree of variance exists within this population. Despite our study including the largest reported number of type I patients to date, it remains difficult to draw firm conclusions from only 30 patients. Only two clinical trials, SUPAP (NCT00541008) and RAPTOR (NCT00688753), have enrolled pRCC patients by subtype, but each enrolled only 15 and 13 type I patients, respectively 19, 20. Metastatic type I tumors appear to be far less common than type II, and are consequently more difficult to study. This may be related to previously reported findings that localized type II tumors often present with higher TNM staging than type I tumors 21, 22, 23.

The activity of targeted therapies in pRCC has varied between studies, but in general, outcomes are thought to be better in metastatic ccRCC than in metastatic pRCC 9. Our results demonstrate that ccRCC patients had better OS, PFS, and RR when treated with targeted therapy than pRCC patients. Within pRCC, no survival differences were detected between VEGF agents and mTOR agents, however VEGF therapies had a more favorable ORR. Although difficult to compare across studies due to heterogeneous patient populations, ORR to sunitinib has been previously reported to range between 0–24%, with the exception of one Korean study in which the ORR was 36% 14, 24, 25, 26, 27. ORR to everolimus has yet to surpass 7% 27, 28, 29. In our analysis, the majority of mTOR‐treated patients received temsirolimus rather than everolimus. This was likely due to the influence of a subgroup analysis of a phase III trial that reported an improved clinical benefit of temsirolimus over interferon alpha in patients with nonclear histology 17.

Despite mTOR inhibitors potentially having a lower ORR than VEGF inhibitors in pRCC patients, studies to date have not been powered to detect a difference in survival outcomes. Two recent randomized phase II clinical trials tested first‐line everolimus versus sunitinib in nonclear RCC patients. ASPEN included 70 pRCC patients and found sunitinib to have a higher ORR (24% vs. 5%) and a longer median PFS (8.1 months vs. 5.5 months) than everolimus, but reported no difference in OS 27. The ESPN trial included 27 pRCC patients. Everolimus displayed a median PFS of 4.1 months and a median OS of 14.9 months, both of which were not superior to sunitinib, which had a median PFS of 5.7 months and a median OS of 16.6 months 24. In our study the 23 patients receiving everolimus reached higher OS (26 months) than patients being treated with sunitinib (15 months) but no robust statements can be made given the small numbers of patients treated with everolimus.

Only two phase II trials have been conducted that specifically limited enrollment to pRCC patients and analyzed by subtype. The SUPAP trial studied first‐line sunitinib in 15 type I and 46 type II pRCC patients, while the RAPTOR trial has reported interim per protocol results on 13 type I and 40 type II patients treated with first‐line everolimus. SUPAP reported similar ORRs to sunitinib, 11% and 13% for type I and type II, respectively. Median PFS was 6.6 months (95% CI 2.8–14.8) in type I and 5.5 months (95% CI 3.8–7.1) in type II 19. Median OS was 17.8 months (95% CI 5.7–26.1) for type I and 12.4 months (95% CI 8.2–14.3) for type II. Treatment with everolimus in the RAPTOR trial has so far resulted in a median PFS of 6.2 months (95% CI 2.1–9.9) for type I and 3.7 months (95% CI 1.9–5.7) for type II 20. Median OS was 28.0 months (95% CI 4.0–28.0) in type I and 20.0 months (95% CI 11.0‐NR) in type II 20.

While sunitinib was the most predominately used targeted therapy in this study, it is important to note that the data collection period spans from 2004–2016, thus trends will inevitably shift based on the approval of additional agents. Of note are several upcoming clinical trials focusing exclusively on pRCC patients. These include the final results of the phase II RAPTOR trial (first‐line everolimus monotherapy; NCT00688753), the ongoing phase II AXIPAP trial (first‐line axitinib; NCT02489695), and the ongoing PAPMET trial (NCT02761057). The PAPMET trial is a large, randomized phase II trial comparing four therapies: sunitinib, cabozantinib, crizotinib, and volitinib, for which the latter three target the *MET* pathway. *MET* pathway alterations were traditionally associated with type I pRCC, however recent studies have identified that type II pRCCs can also display high‐*MET* expression 4, 5. The rationale for targeting *MET* in pRCC was previously demonstrated in a phase II trial with foretinib, a dual MET/VEGFR2 inhibitor, that produced an RR of 13.5% and a median PFS of 9.3 months 30. Volitinib's targeting of *MET* has also demonstrated potential utilization according to preliminary phase I/II trial results (NCT02127710); a phase III trial is planned 31. Additionally, it is likely that PD‐L1 inhibitors will be tested in pRCC in the near future, given evidence that ~10% of pRCC tumors express PD‐L1, and these patients appear to have worse outcomes than PD‐L1 negative tumors 32.

In summary, there is no current standard of care for metastatic pRCC as the evidence to guide treatment decisions is limited by small sample sizes. This is the largest analysis of metastatic pRCC and its subtypes to date. Our analysis is limited by its retrospective design and the lack of a centralized review, however such review was not feasible and our data is more reflective of standard daily practice. In addition, the authors acknowledge that subtyping of pRCC is becoming less dependent on histologic review but is instead shifting towards genetic analysis, which may explain some of the variance in subtype outcomes 4.

## Conclusion

This study provides more evidence that metastatic pRCC has worse OS, PFS, and RR outcomes as compared to ccRCC when treated with contemporary targeted therapy. In this dataset, type II pRCC was over five times more common than type I pRCC in metastatic disease. We found that type I pRCC outcomes were similar to ccRCC however a great deal of variance exists in this subgroup, preventing a firm conclusion that metastatic type I pRCC has a better prognosis than metastatic type II pRCC. Importantly, the IMDC prognostic model was effective in stratifying patients into favorable‐, intermediate‐, and poor‐risk outcomes. Upcoming clinical trials may provide better guidance on management of pRCC patients but until better therapies are established, it is reasonable to offer pRCC patients targeted therapy.

## Conflict of Interest

None declared.
